# Development, organization and plasticity of auditory circuits: Lessons from a cherished colleague

**DOI:** 10.1111/ejn.13979

**Published:** 2018-08-16

**Authors:** Michael Lohse, Victoria M. Bajo, Andrew J. King

**Affiliations:** ^1^ Department of Physiology, Anatomy and Genetics University of Oxford Oxford UK

**Keywords:** cortex, corticocollicular, corticothalamic, ferret, midbrain, thalamus

## Abstract

Ray Guillery was a neuroscientist known primarily for his ground‐breaking studies on the development of the visual pathways and subsequently on the nature of thalamocortical processing loops. The legacy of his work, however, extends well beyond the visual system. Thanks to Ray Guillery's pioneering anatomical studies, the ferret has become a widely used animal model for investigating the development and plasticity of sensory processing. This includes our own work on the auditory system, where experiments in ferrets have revealed the role of sensory experience during development in shaping the neural circuits responsible for sound localization, as well as the capacity of the mature brain to adapt to changes in inputs resulting from hearing loss. Our research has also built on Ray Guillery's ideas about the possible functions of the massive descending projections that link sensory areas of the cerebral cortex to the thalamus and other subcortical targets, by demonstrating a role for corticothalamic feedback in the perception of complex sounds and for corticollicular projection neurons in learning to accommodate altered auditory spatial cues. Finally, his insights into the organization and functions of transthalamic corticocortical connections have inspired a raft of research, including by our own laboratory, which has attempted to identify how information flows through the thalamus.

## Introduction

During his two spells at the University of Oxford, Ray Guillery was a greatly valued colleague, who showed a close interest in our research on the auditory system. However, his influence extended well beyond interactions at seminars and by email. From his pioneering work on the ferret as a model for sensory development to his insights into the role of descending corticofugal projections, and particularly the connections and circuitry of the thalamus, Ray had a profound impact on the direction of our research. In this review, we set out the various ways in which the work of our group has been guided over a period of more than 30 years by his many contributions. We place these interactions in the context of our current understanding of some key areas of auditory system development and plasticity, and of the perceptual and behavioural consequences of corticofugal modulation on processing in the thalamus and midbrain.

## Animal models of sensory development

One of Ray Guillery's most well‐known early achievements was to characterize the abnormality in the retinogeniculate pathway of Siamese cats, in which some of the axons that target the thalamus are misrouted to the wrong side of the brain (Guillery, Casagrande & Oberdorfer, 1974). This led to his more general interest in the developmental changes produced in the visual system of albino animals, of which Siamese cats are an example. Although Ray Guillery's work spanned a range of species, from axolotls to humans, prominent among them were ferrets, which he opted to use both because albinos are easily obtained and because he recognized the particular advantages afforded by this species for studying brain development. These advantages include relatively large litters and an unusually short gestation period, meaning that various aspects of development that take place prenatally in primates and other carnivores are delayed until after birth in ferrets. In particular, the eyes naturally remain closed until approximately 30 days after birth, which is also near the age at which the onset of hearing occurs (Moore, 1982). This therefore provides a broad postnatal window for studying the events leading up to the stage at which sensory processing begins, as well as for examining the age‐dependent consequences of manipulating peripheral inputs on neural circuit maturation, without the need for difficult in utero surgery.

Following Ray Guillery's initial observations of a reduced uncrossed projection from the eye to the thalamus in albino ferrets (Guillery, 1971a), he and his co‐workers carried out an anatomical study of the postnatal development of the retinogeniculate pathways in normally pigmented ferrets (Linden, Guillery & Cucchiaro, 1981). This study led to several other groups adopting this species as a model for investigating the organization, development and plasticity of the visual system (reviewed in Sharma & Sur, 2014), often initially as an alternative to the previously well‐studied cat, but also because its visual system is more advanced than that of rodents. Indeed, the primary visual cortex (V1) in ferrets contains columnar maps of stimulus features, such as orientation selectivity (White, Bosking & Fitzpatrick, 2001), that are typical of primates and other carnivores (Nauhaus, Benucci, Carandini & Ringach, 2008) and usually thought to be lacking in rodents (Ohki, Chung, Ch'ng, Kara & Reid, 2005). Consequently, the ferret is widely regarded as a good model for studying the functional organization of cortical circuits and their maturation.

### Ferrets in Oxford

After moving to Oxford to become Head of the Department of Human Anatomy in 1984, Ray Guillery continued to use ferrets to explore the rules governing the development of the visual pathways and particularly the basis for the abnormalities that result from the albino gene. At that time, ferrets were already becoming a popular model for studying sensory systems in several research groups in the nearby University Laboratory of Physiology. Both Zaineb Henderson and Ian Thompson were working on the visual system in this species, while David Moore had started to use ferrets to study auditory system development. In the years that followed, Ian Thompson's research on retinal ganglion cells (Wingate & Thompson, 1995), retinofugal decussation patterns (Morgan, Henderson & Thompson, 1987; Thompson & Morgan, 1993) and thalamocortical processing in adult and developing ferrets (e.g., Akerman, Grubb & Thompson, 2004; Akerman, Tolhurst, Morgan, Baker & Thompson, 2003; Baker, Thompson, Krug, Smyth & Tolhurst, 1998) built on the work of Ray Guillery and his colleagues, and in several ways, the auditory group's early research also paralleled their work. Indeed, complementary studies were carried out in both departments demonstrating that the anatomical abnormalities resulting from albinism extend to the auditory system (Baker & Guillery, 1989; Moore & Kowalchuk, 1988a). Ferrets have continued to be the principal animal model used by the Oxford auditory neuroscience group, with more than 100 publications so far resulting from this work.

## Experience‐dependent plasticity in developing sensory systems

Ray Guillery made effective use of experimental manipulations that altered visual inputs to provide valuable insights into the nature of the processes involved in the maturation of retinogeniculocortical circuits. For example, his earlier studies on the effects of monocular deprivation in cats provided compelling evidence that a competitive interaction takes place between each eye during the development of the visual pathways (Guillery & Stelzner, 1970; Sherman, Guillery, Kaas & Sanderson, 1974). Thus, unilateral lid suture leads to an expansion of the thalamocortical projection from the open eye within the binocular region of the visual pathways at the expense of the input from the deprived eye. In a similar vein, the Oxford auditory group undertook a series of experiments in ferrets that set out to investigate the effects of unilateral hearing loss on the morphology, connectivity and response properties of neurons in the brainstem. Among other findings, these studies demonstrated that removal of one cochlea within a developmental sensitive period altered the laterality of the projections from the cochlear nucleus (CN), the first stage of central auditory processing, to the inferior colliculus (IC) in the midbrain (Moore & Kowalchuk, 1988b). Specifically, this manipulation resulted in an increase in the number of neurons in the CN on the opposite side of the brainstem that project to the ipsilateral IC. A comparable, albeit less pronounced, rewiring of this pathway was also observed after plugging one ear in juvenile ferrets, which was interpreted in a similar way to the work from Ray Guillery's laboratory as evidence for competition between the two ears for synaptic space on binaurally innervated neurons in nuclei such as the IC (Moore, Hutchings, King & Kowalchuk, 1989).

Understanding the functional consequences of a change in the balance of activity between the eyes or ears is obviously very important. Moving beyond their predominantly anatomical approach, Ray Guillery and his colleagues, particularly Murray Sherman, adopted behavioural and electrophysiological measures to show in cats that responses to stimulation of a previously deprived eye are largely eliminated within the binocular regions of the visual pathways (Sherman et al., 1974). A related result was obtained by McAlpine, Martin, Mossop and Moore (1997), who found that monaural deafening in young ferrets led to an increase in the proportion and responsiveness of neurons in the contralateral midbrain that were activated by the intact ear. Moreover, several years later, Popescu and Polley (2010) reported that inducing a reversible conductive hearing loss in one ear in developing rats weakened the representation of that ear in the IC and even more so in the primary auditory cortex (A1), whereas the representation of the non‐deprived ear became strengthened. The physiological changes that occur following unilateral hearing loss therefore again support the concept of competitive interactions between the two sense organs in both the visual and auditory systems.

The plasticity resulting from an imbalance in inputs between the two eyes or ears during early life is often regarded as maladaptive because the changes induced in the brain can disrupt the capacity of neurons to integrate signals from each pair of sense organs and may outlast the period of sensory deprivation, giving rise to amblyopia—impaired visual acuity in an otherwise normal eye—and its auditory equivalent amblyaudia (Kaplan et al., 2016). However, adaptive changes can also take place in the brain following sensory deprivation that serve to compensate for the abnormal inputs experienced during development. This has been demonstrated most clearly in the context of sound localization, which relies on the sensitivity of the auditory system to physical cues arising from the geometry of the head and external ears (King, Schnupp & Doubell, 2001). Because of the physical separation of the ears on either side of the head, sound originating from one side will arrive at the closer ear first, giving rise to interaural time differences (ITDs) that vary in magnitude with the horizontal direction of the sound source relative to the head. Depending on the frequency composition of the sound, interaural level differences (ILDs) may also be produced because of the acoustic shadow cast by the head, while interactions with the folds of the external ears can modify the amplitude spectrum of the sound in a direction‐dependent fashion. The cue values corresponding to each direction in space depend on the size, shape and separation of the ears and therefore change naturally over the course of development as the head and ears grow (Campbell et al., 2008; Schnupp, Booth & King, 2003). This implies that the developing neural circuits have to be plastic to allow them to accommodate the cues experienced by each individual.

**Figure 1:**
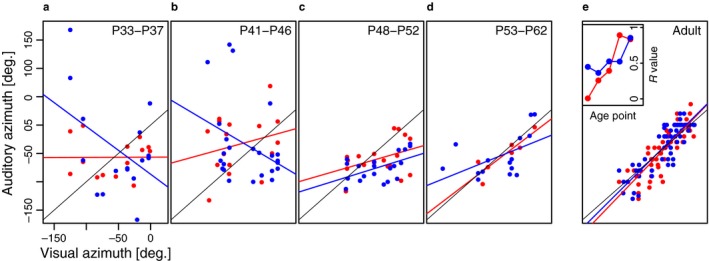
Maturation of auditory spatial topography in the ferret SC. (a–e) Each panel plots the auditory best azimuth of deep layer SC units as a function of the visual best azimuth of multiunit activity recorded in the overlying superficial layers. The data were obtained at the postnatal ages indicated at the top of each panel using free‐field stimuli. Because adult‐like visual topography is present in the superficial SC layers throughout this developmental period, the topographic order of the auditory map is reflected in the degree to which it is in register with the visual map. Auditory responses recorded at near‐threshold sound levels (approximately 10 dB > unit threshold) are shown in red and suprathreshold levels (approximately 25 dB > unit threshold) in blue. A linear regression was fitted to the data from each sound level at each age group (red and blue lines; the black line is the 45° diagonal indicating perfect alignment of the visual and auditory data). The inset panel in (e) plots the correlation coefficient (*R*) of each regression slope as a function of age. At both sound levels, there is a steady increase in the *R* value during development, indicating an improvement in topographic order in the auditory representation. From Campbell et al. (2008) with permission.

**Figure 2:**
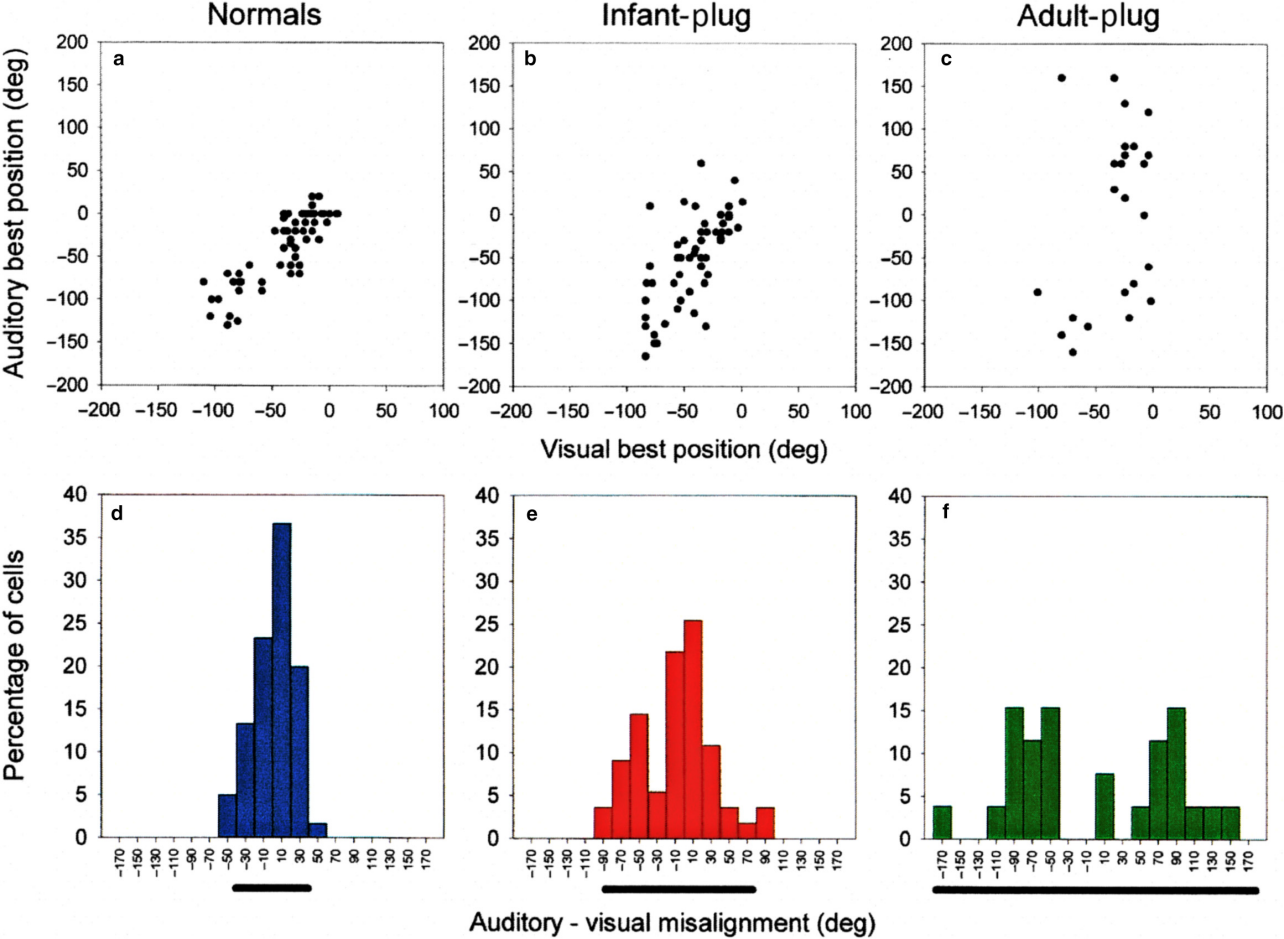
Effects of chronic monaural occlusion on the registration of the auditory and visual maps in the ferret SC. Each panel shows the relationship between the representations of visual azimuth in the superficial layers and auditory azimuth in the deeper layers of the SC. (a–c) Recordings were made from anesthetized ferrets, and for each vertical electrode penetration, the visual best azimuth is plotted against the auditory best azimuth (measured with 100‐ms broadband noise bursts at sound levels of 25–35 dB above unit threshold). deg, degree. (d–f) The frequency histograms plot the angular difference between the visual and auditory best azimuths; the bar below each histogram is centred on the mean misalignment and extends to 2 SDs on either side. (a and d) Data from normal, adult ferrets. (b and e) Data from adult ferrets that had been raised from just before the onset of hearing (which in ferrets occurs ≈4 weeks after birth) with the ear ipsilateral to the recording site occluded. (c and f) Data from adult ferrets that had one ear plugged for a comparable period, this time beginning when they were at least 6 months old. The data shown in b, c, e and f were obtained with the earplug still in place. F tests revealed that the variance in auditory‐visual misalignment is significantly different between each of the three groups. Because the superficial layer visual map showed a high degree of topographic order in each case, the increased scatter in the relationship between the two maps in the plugged animals is indicative of poorer topographic order in the auditory representation. These comparisons indicate some adaptation to the altered cues in the ferrets that were raised with one ear occluded but not in the ferrets that were plugged as adults. From King et al. (2000) with permission.

Interest in the experience‐dependent plasticity of auditory spatial processing focussed initially on the superior colliculus (SC), which, in contrast to the lemniscal auditory pathway, represents sound‐source direction topographically (Palmer & King, 1982). The Oxford auditory group found that the auditory spatial receptive fields of ferret SC neurons become more sharply tuned and that topographic order emerges gradually during the course of postnatal development (Campbell et al., 2008) (Figure 1). While the sharpening of the receptive fields can be accounted for by the growth‐related changes in the localization cues that take place over this period, the process of aligning the developing auditory map with the representation of other sensory modalities relies on experience. Building on seminal studies carried out in barn owls (e.g., Knudsen, 1985), recordings from ferrets raised with one ear occluded revealed a near‐normal map of auditory space, indicating that the developing auditory system had compensated for the altered localization cues values available. In contrast, this work failed to show comparable plasticity of auditory spatial tuning in the SC of adult ear‐plugged ferrets (King, Hutchings, Moore & Blakemore, 1988; King, Parsons & Moore, 2000) (Figure 2).

Adaptive changes in auditory spatial processing in the developing SC have also been demonstrated by manipulating visual inputs. For instance, shifting the visual world representation relative to the head either optically, as in prism‐rearing experiments in barn owls (Knudsen & Brainard, 1991), or surgically in ferrets (King et al., 1988) was found to produce a corresponding shift in auditory spatial selectivity in the SC, even though the auditory localization cues were unchanged. These studies suggest that visual inputs, which generally provide more precise and reliable spatial information, might provide a template for guiding the development of the auditory responses in the SC (King, Schnupp & Thompson, 1998), so that multisensory signals arising from the same object or event can interact to guide orientation behaviour (Wallace, Perrault, Hairston & Stein, 2004).

While the use of ferrets by a number of research groups to study the development and plasticity of sensory systems owes much to Ray Guillery's pioneering work in this species, the majority of these studies were initially restricted to anatomical and electrophysiological approaches. As previous work in cats and monkeys had illustrated, however, it was clear that behavioural methods would be needed to assess the functional significance of the plasticity demonstrated in the processing of sensory information. Fortunately, it turned out that ferrets can be readily trained to carry out sensory tasks. This species is increasingly being used to study aspects of visual (Garipis & Hoffmann, 2003; Von Melchner, Pallas & Sur, 2000 *;* Zhou, Yu, Sellers & Fröhlich, 2016) and multisensory behaviour (Hammond‐Kenny, Bajo, King & Nodal, 2017; Hollensteiner, Pieper, Engler, König & Engel, 2015), and has been employed extensively in a range of auditory detection, discrimination and localization tasks (reviewed by Fritz, Elhilali, David & Shamma, 2007; Nodal & King, 2014).

**Figure 3:**
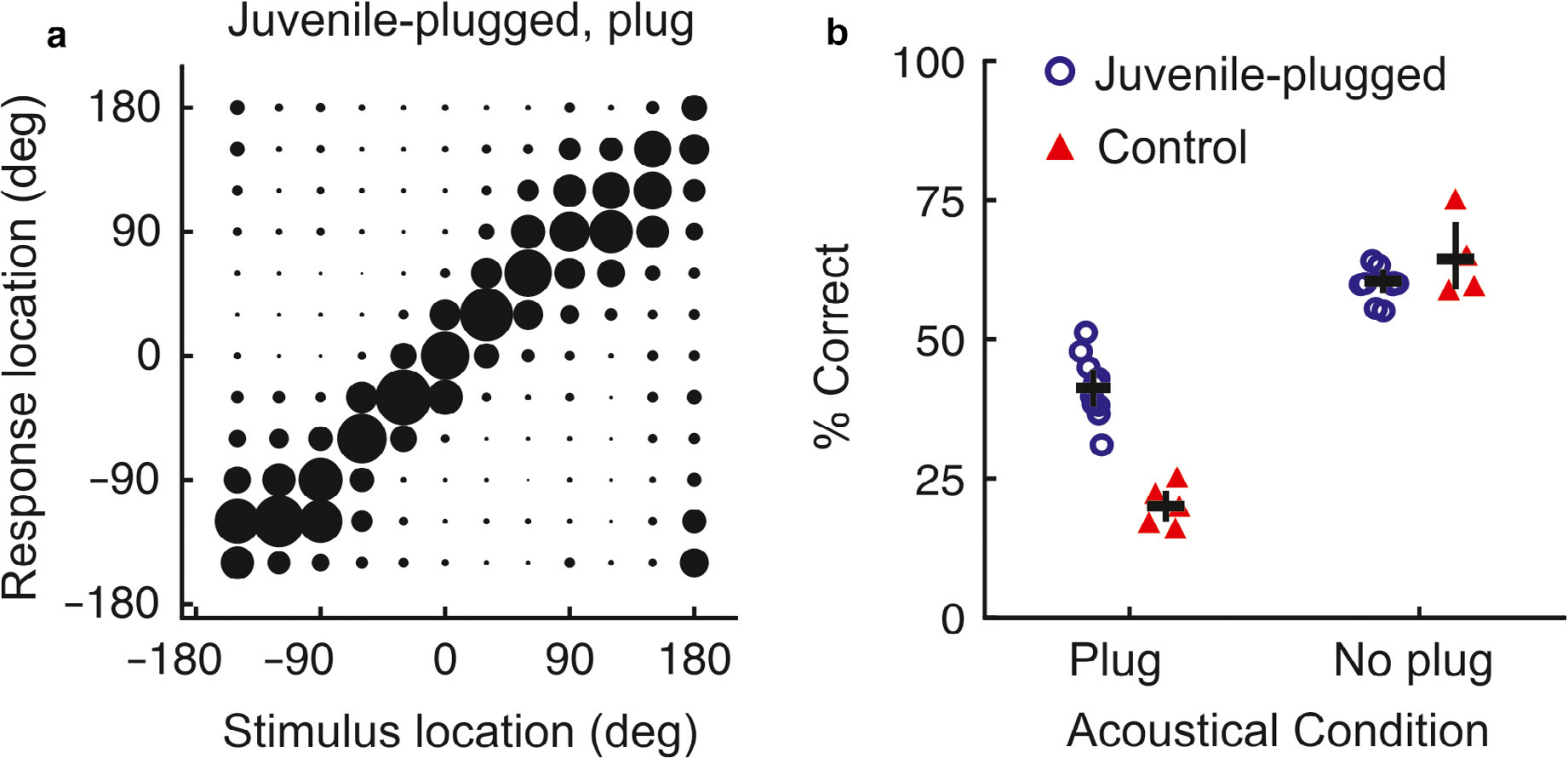
Adaptive changes in sound localization behaviour following developmental hearing loss in one ear. (a) Average joint distributions of stimulus and response location for ferrets raised with one ear occluded and tested with the earplug in place; the size of the circles represents the proportion of trials for each stimulus–response combination. The stimuli were 200‐ms duration broadband noise bursts. (b) Percentage correct scores for juvenile‐plugged and control groups, with individual animals denoted by symbols, tested either with (“Plug”) or without (“No plug”) an earplug in one ear. Horizontal lines indicate mean values, with error bars showing bootstrapped 95% confidence intervals. Adapted with permission from Keating et al. (2013).

Building on the anatomical and physiological data described above, our laboratory initially focussed on the effects of raising ferrets with a unilateral conductive hearing loss on various measures of spatial hearing. Long‐term occlusion of one ear, initiated either during infancy or in adulthood, was found to reduce the ability of ferrets to detect a tone in the presence of masking noise originating from other directions, with levels of “binaural unmasking” gradually recovering after normal binaural inputs were restored (Hine, Martin & Moore, 1994; Moore et al., 1999). This result therefore seemed to add to the evidence that monaural hearing loss can impair the representation of that ear in the brain. However, in keeping with our SC recordings, evidence for adaptive plasticity in monaurally deprived ferrets emerged when a free‐field sound localization task was used (Keating, Dahmen & King, 2013, 2015; King et al., 2000). In this task, animals were trained by positive conditioning to initiate a trial by licking a spout positioned in front of a central start platform, which triggered the presentation of a noise burst from one of 12 loudspeakers positioned at 30° intervals around the perimeter of the testing chamber. The performance of the animals was assessed as the duration, level and spectral composition of the stimulus were varied, by measuring both the accuracy and latency of the initial head‐orienting response made following sound presentation and the loudspeaker/reward spout subsequently approached. Plugging one ear changes the binaural ILDs and ITDs corresponding to each direction in space and initially produced substantial localization errors. However, ferrets raised with an earplug placed in one ear and tested with that ear still occluded were able to localize sound reasonably accurately, indicating that the developing brain can adapt to a substantial degree to an imbalance in inputs between the two ears (Keating et al., 2013, 2015; King et al., 2000) (Figure 3).

**Figure 4:**
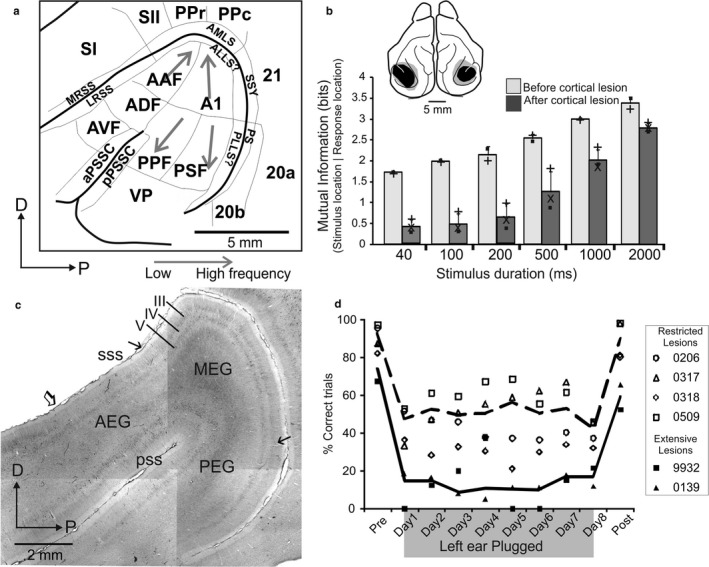
The ferret auditory cortex and its role in sound localization behaviour. (a) Different areas identified in and surrounding the ectosylvian gyrus; see main text (modified with permission from Bizley et al., 2015). Grey arrows indicate the direction of tonotopic gradients (i.e., low to high frequency). (b) Effect of lesions of the ectosylvian gyrus on the ability of ferrets to localize broadband noise bursts of different duration. Performance is indicated by the mutual information between the target and response locations; these values were significantly lower following the auditory cortical lesions, indicating poorer performance (modified with permission from Nodal et al., 2010). (c) Picture of a flattened section at the level of the ectosylvian gyrus showing the principal regions (MEG, AEG and PEG) immunostained with the SMI_32_ antibody. The bilaminar staining pattern of pyramidal cells in layers III and V defines MEG (modified with permission from Bajo et al., 2007). (d) In contrast to controls (not shown), animals with lesions of the auditory cortex (either restricted to the primary fields in MEG or including more extensive regions of the ectosylvian gyrus) show no recovery in their sound localization behaviour following the initial impairment in performance caused by plugging one ear (modified with permission from Nodal et al., 2010).

This behavioural plasticity closely matches the adaptive changes previously observed in the auditory spatial tuning of SC neurons in monaurally deprived ferrets (King et al., 1988, 2000). However, in one of the first auditory behavioural studies in ferrets, Kavanagh and Kelly (1987) had shown that aspiration lesions of the auditory cortex, including those restricted to A1, disrupt the accuracy of approach‐to‐target responses in a 2‐loudspeaker version of the sound localization we had used. We subsequently confirmed this result for our 12‐loudspeaker task in animals in which the cortex was either lesioned (Nodal et al., 2010) (Figure 4a,b) or reversibly deactivated (Nodal, Bajo & King, 2012) and therefore turned our attention to investigating the role of auditory cortical circuits in adaptive plasticity.

## The ferret auditory cortex and adaptive plasticity

Ferrets are a good model for studying hearing, not only because of their particular suitability for developmental and behavioural studies but also because their audible frequency range entirely overlaps, and extends beyond, that of humans (Kelly, Kavanagh & Dalton, 1986). This means that they can be used for investigating auditory functions, such as pitch perception (Walker, Schnupp, Hart‐Schnupp, King & Bizley, 2009; Yin, Fritz & Shamma, 2010) and ITD processing (Keating, Nodal & King, 2014), that rely on low‐frequency hearing. One disadvantage, however, is that many areas of the auditory pathway, including the cortex, have been much less well studied than in other commonly used species, such as cats or macaque monkeys. This is now changing and, like other species, a number of different auditory cortical fields with distinct functional properties have been described in the ferret, with the primary areas, A1 and the anterior auditory field (AAF), located in the middle ectosylvian gyrus (MEG) (Bizley, Nodal, Nelken & King, 2005; Kowalski, Versnel & Shamma, 1995; Nelken et al., 2004; Phillips, Judge & Kelly, 1988) (Figure 4a). Neurons in A1 and AAF have relatively short latency responses and are arranged to form tonotopic maps. Unusually, the tonotopic axes of these fields are arranged in parallel, rather than in opposing directions with a common high‐frequency border, which means that it is difficult to distinguish between them.

Additional acoustically responsive areas were described more ventrally in the anterior and posterior ectosylvian gyrus (AEG and PEG, respectively) using 2‐deoxyglucose autoradiography (Wallace, Roeda & Harper, 1997), with patterns of corticocortical connectivity suggesting that these represent higher‐level areas that receive at least some of their input from the primary areas in the MEG (Bizley, Bajo, Nodal & King, 2015; Pallas & Sur, 1993). The subdivision of the ectosylvian gyrus into these three main regions—MEG, AEG and PEG—is also supported by patterns of staining for cytochrome oxidase activity or using antibodies against the neurofilament protein SMI_32_ (Figure 4c). Two tonotopically organized cortical areas, the posterior suprasylvian field (PSF) and the posterior pseudosylvian field (PPF), are found on the PEG, which share a common low‐frequency border with A1, with the neurons found there displaying distinct temporal firing patterns from those located in the primary fields (Bizley et al., 2005) (Figure 4a). Electrophysiological mapping studies in our laboratory also documented two areas, the anterodorsal field (ADF) and the anteroventral field (AVF), in the AEG, where neurons respond to sound but lack the tonotopic order previously described for the other areas (Bizley et al., 2005) (Figure 4a). There is also anatomical and electrophysiological evidence for additional, more ventral PEG areas (Atiani et al., 2014; Bajo, Nodal, Bizley, Moore & King, 2007; Bizley et al., 2015; Pallas & Sur, 1993), but these have yet to be fully characterized, while the presence of neurons within the ectosylvian gyrus whose activity is modulated by other sensory modalities has provided further insights into the functional organization of this part of the ferret brain (e.g., Bizley, Nodal, Bajo, Nelken & King, 2007; Manger, Engler, Moll & Engel, 2005; Ramsay & Meredith, 2004).

In view of the importance of auditory cortex for normal sound localization accuracy and the considerable evidence for experience‐dependent plasticity in the response properties of its neurons (Dahmen & King, 2007; Popescu & Polley, 2010), our group set out to investigate the involvement of A1 in adaptation to hearing loss in one ear during development. This required determining the basis by which the animals adapt. Ferrets raised with an earplug in one ear developed the ability to localize broadband sounds accurately by becoming more dependent on the unchanged spectral localization cues provided by the contralateral ear (Keating et al., 2013). Although seemingly at odds with the basis for adaptation to monaural hearing loss reported in developing barn owls (Mogdans & Knudsen, 1992), experience‐dependent reweighting of different auditory spatial cues has been observed in humans (Keating, Rosenior‐Patten, Dahmen, Bell & King, 2016). Furthermore, the behavioural plasticity present in monaurally deprived ferrets was paralleled by changes in neuronal responses in A1, which showed increased sensitivity to the monaural spectral cues provided by the non‐occluded external ear (Keating et al., 2013). Interestingly, this cue reweighting was found to be rapidly reversible, as the behavioural and physiological data obtained from ferrets raised with one ear occluded both showed a reduced dependence on spectral cues when the earplug was removed. Subsequent measurements in the same animals revealed that a compensatory adjustment in ILD sensitivity had also taken place (Keating et al., 2015), with largely separate populations of A1 neurons showing adaptive plasticity in the processing of monaural spectral cues and binaural cues (Keating et al., 2016).

In search of a potential sensitive or critical period for the plasticity produced by monaural deprivation, we unexpectedly found that normally raised, adult ferrets can rapidly recover their ability to localize sound with one ear occluded, with the extent and rate of adaptation being determined by how often they are trained (Kacelnik, Nodal, Parsons & King, 2006). Much less plasticity was seen in animals in which the auditory cortex was lesioned (Nodal et al., 2010) (Figure 4d) or deactivated pharmacologically (Nodal et al., 2012), with learning impaired after silencing both primary and higher‐level cortical fields. Critically, this disruptive effect on adaptation was observed using stimulus durations at which there was no change in localization accuracy under normal hearing conditions and therefore did not reflect a reduced ability to perform the task. Adaptation in monaurally deprived ferrets is also impaired if cholinergic inputs to the cortex from the basal forebrain are removed (Leach, Nodal, Cordery, King & Bajo, 2013), suggesting that cholinergic modulation of cortical responses contributes to sensory processing under challenging listening conditions, such as those experienced when different localization cues provide conflicting information. Together, these studies confirm that the auditory cortex plays a critical role in spatial hearing and in the experience‐dependent plasticity that allows the brain to compensate for asymmetric reversible hearing loss. This is only part of the story, however, and an equally important question, which we shall consider in the following section, is how cortical activity affects other levels of sensory processing, particularly in subcortical nuclei.

## Descending corticofugal projections

Another of the major contributions of Ray Guillery, again working with Murray Sherman, to directly influence our own research was their exploration of the connections between the thalamus and the cortex. Information flowing from the peripheral sense organs towards the cortex has received disproportionately more attention relative to the information flowing in the opposing direction (Sherman & Guillery, 2001), and a fundamental question, which they set out to address, is how corticothalamic projections contribute to thalamocortical processing. This has led to a surge in interest in the role in perception and behaviour of descending corticofugal projections in general and of the thalamus in particular.

**Figure 5:**
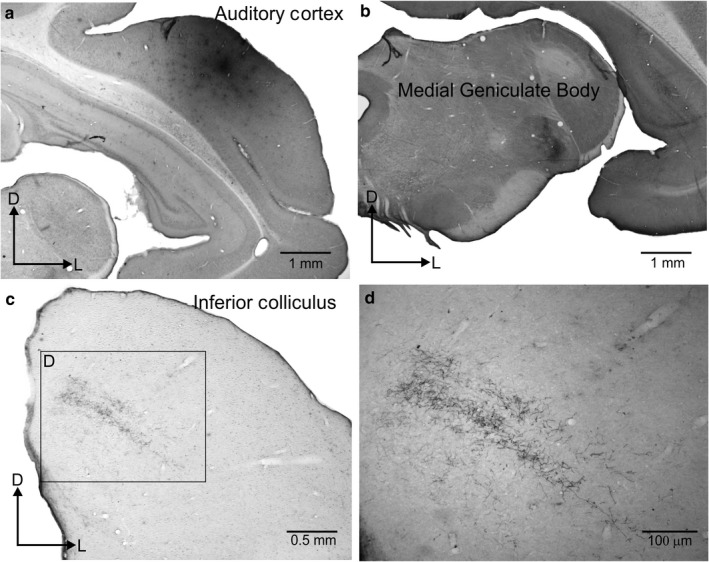
Descending projections in the ferret from the auditory cortex to the medial geniculate body in the thalamus and the inferior colliculus in the midbrain. (a) Injection site of rhodamine (fluororuby) in the primary auditory cortex. (b) Terminal fields in the MGB are mainly located in the ventral division with a dorsoventral orientated strip that follows the MGB external curvature. (c) Terminal fields in the IC, including dorsal cortex and dorsal part of the central nucleus, and extending with the same dorsomedial to ventrolateral orientation as the IC fibro‐dendritic laminae. (d) Boxed area from c taken at higher magnification.

Descending corticofugal pathways are, however, not restricted to the thalamus. In the auditory system, these projections are unusually extensive, reaching almost every processing level, particularly the IC (Figure 5), but also the SC (Bajo, Nodal, Bizley & King, 2010), paralemniscal areas, periolivary regions of the superior olivary complex (SOC) and the CN (reviewed in Winer, 2005). Outside the auditory system, descending projections from the auditory cortex innervate the amygdala (Romanski & LeDoux, 1993), striatum and pontine nuclei (Perales, Winer & Prieto, 2006). Neurons in cortical layers V and VI are the source of these descending projections and there is growing evidence that the projection neurons in these output layers differ in their morphology, connections and physiological properties, implying that they make distinct contributions to information processing in their subcortical targets (Sherman & Guillery, 2001).

In the following sections, we consider possible functions of the corticofugal projections originating in layers V and VI, with the principles established by Sherman and Guillery in mind, but with a focus on recent studies by our own and other groups in the auditory system.

### Cortical layer VI feedback projections

Layer VI pyramidal cells predominantly target first‐order nuclei in the sensory thalamus, such as the dorsal division of the lateral geniculate nucleus (LGN), ventral division of the medial geniculate body (MGBv) or the ventral posterior nucleus (VPN). An important revelation about the likely significance of these descending projections came from the finding that they provide a very large proportion of the synapses found on thalamic relay neurons (Guillery, 1969, 1971b; Sherman & Koch, 1986). Indeed, studies in the visual system have revealed that only around 10% or less of the synapses on these relay neurons come from their ascending sensory inputs, with the rest originating predominantly from the cortex, ventral thalamus or brainstem (Guillery & Sherman, 2011; Van Horn, Erişir & Sherman, 2000). However, layer VI corticothalamic synapses are small and have been shown to have a modulatory rather than a driving effect on neurons in the thalamus, suggesting that they convey feedback signals from the cortex that influence the subcortical processing of sensory information (Sherman & Guillery, 2001). Measurements of the postsynaptic responses evoked by activation of corticothalamic synapses indicate that these signals are likely to have a pronounced effect on the transmission of information through the thalamus (Bartlett & Smith, 2002; McCormick & von Krosigk, 1992; Turner & Salt, 1998; Von Krosigk, Monckton, Reiner & McCormick, 1999). Furthermore, layer VI neurons can have a pronounced modulatory influence on the activity of neurons in other cortical layers, suggesting that there are multiple sites at which they can influence thalamocortical processing (Bortone, Olsen & Scanziani, 2014; Guo, Clause, Barth‐Maron & Polley, 2017; Lee, Lam & Sherman, 2012; Olsen, Bortone, Adesnik & Scanziani, 2012).

In addition to providing direct excitatory inputs to thalamocortical relay neurons, the axons of layer VI neurons branch to terminate on GABAergic neurons in the thalamic reticular nucleus (TRN). This dual termination provides a way for the cortex to modulate the activity of thalamic relay cells in diverse ways, using a combination of excitation and inhibition. A recent example of the dynamic effects of this descending projection comes from a study by Crandall, Cruikshank and Connors (2015), showing that whether layer VI corticothalamic neurons facilitate or suppress activity in the medial part of the VPN depended on their frequency of firing, as well as how long this excitation pattern was maintained. This dynamic switch is made possible by the interactions between the direct excitatory projection to the VPN neurons and their inhibitory disynaptic inputs provided via the TRN (Crandall et al., 2015).

A recent study provided a beautiful example of how dynamic modulation of thalamic and cortical circuits can promote certain perceptual abilities over others. Investigating the role of layer VI corticothalamic neurons in the mouse auditory system, Guo et al. (2017) found that at short delays following optogenetic activation of these neurons, sound‐evoked responses were suppressed in A1, but not in MGBv, whereas, at longer delays, both cortical and thalamic responses were enhanced. They also showed that this initial intracortically induced suppression increased performance on a sound frequency discrimination task, at the expense of detection performance, while the subsequent increase in excitability in the thalamocortical system was associated with better detection performance but poorer discrimination. Such studies demonstrate how contemporary methods for dissecting neural circuitry are enabling Ray Guillery's early ideas about the role of layer VI corticothalamic feedback to be investigated in the behaving animal.

**Figure 6:**
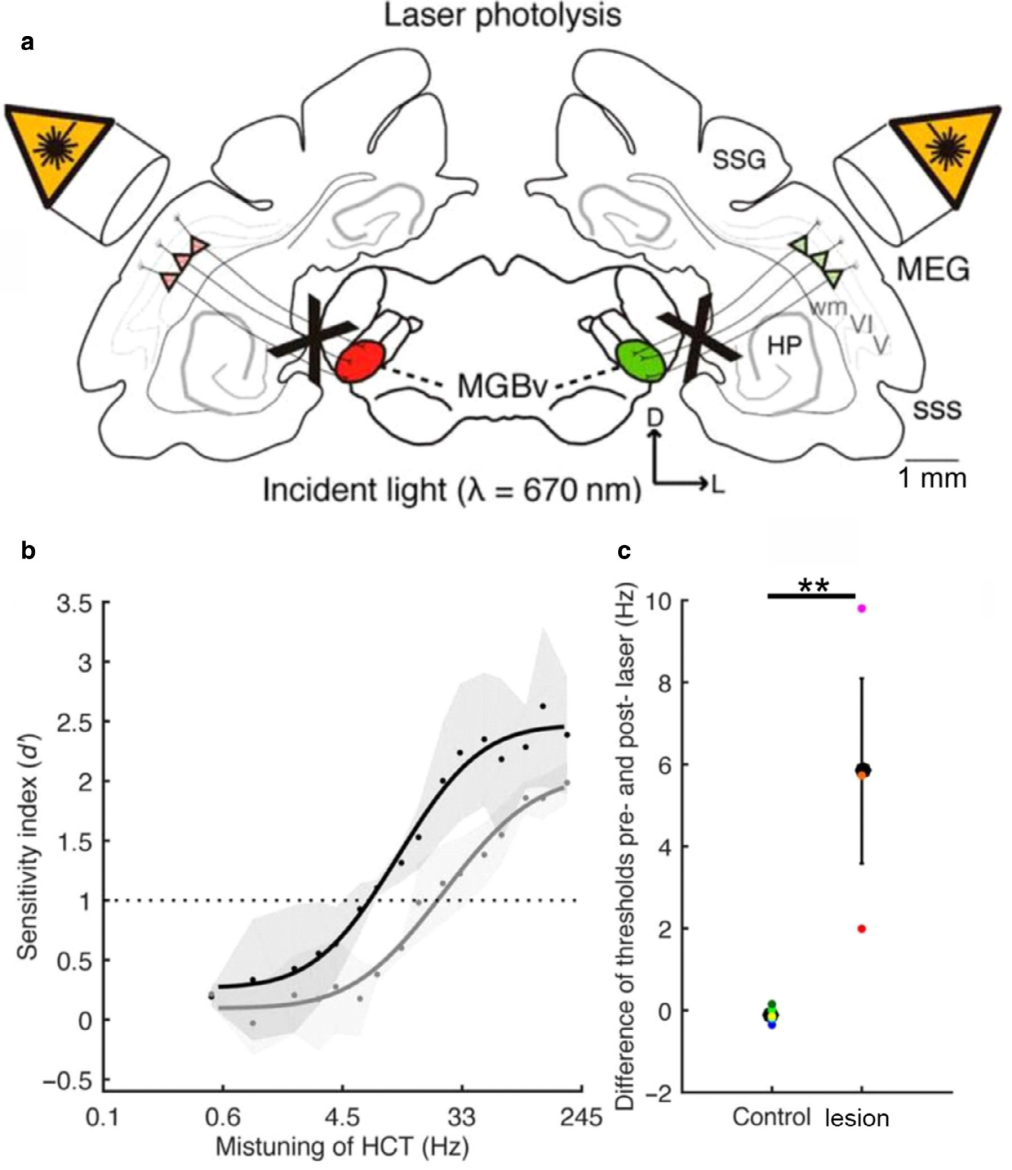
Mistuning detection performance is impaired after ablation of the A1‐MGBv feedback projection. (a) Schematic of chromophore‐targeted laser photolysis method. Red and green conjugated fluorescent retrobeads were injected in each MGB, followed some weeks later by exposing A1 bilaterally to near‐infrared laser light to induce apoptosis of layer VI corticothalamic projection neurons. In this figure, V and VI refer to cortical layers. (b) Mistuning sensitivity (*d*′) before (black) and after (grey) A1‐MGBv projection was removed. A cumulative Gaussian distribution was used to fit the psychometric functions. Dots represent mean values across animals and grey areas represent the SEM. (c) Threshold differences before and after removal of the A1‐MGBv projection, comparing control and lesion cases (two‐tailed unpaired *t* test, **p <* *0.01). Coloured dots represent data from individual animals. Adapted with permission from Homma et al. (2017).

Our own work has provided another example of the behavioural consequences of manipulating layer VI auditory corticothalamic feedback (Homma et al., 2017) (Figure 6). By injecting fluorescent microbeads conjugated with a light‐sensitive chromophore bilaterally into ferret MGBv, we were able to induce a selective loss of approximately 60% of the retrogradely labelled layer VI neurons in A1 by focusing infrared laser illumination onto this layer. The animals had previously been trained on a go/no‐go task to detect a single mistuned frequency in a complex tone comprising 16 harmonics (Homma, Bajo, Happel, Nodal & King, 2016; Homma et al., 2017). Following the loss of corticothalamic projection neurons, mistuning detection was impaired, as indicated by decreased *d*′ values and a shift of the psychometric curves towards higher mistuning values (Figure 6b,c). This finding suggests that the modulatory influence of A1 feedback on MGBv neurons contributes to auditory scene analysis in ferrets through their ability to perceive the harmonic structure of complex sounds.

Some studies have also described a relatively minor projection from cortical layer VI to the IC, but arising from a different population of neurons with cell bodies located in the deep part of the layer, close to the white matter, which are not always pyramidal in shape (Bajo & Moore, 2005; Coomes, Schofield & Schofield, 2005; Games & Winer, 1988; Schofield, 2009; Slater, Willis & Llano, 2013). Although the physiological properties of these neurons have been measured *in vitro* (Slater et al., 2013), it remains unclear what impact layer VI neurons have on IC processing.

### Cortical layer V feedforward projections

Corticothalamic projections also originate from layer V neurons. In contrast to the layer VI corticothalamic projection, which provides feedback to the same part of the thalamus from which it receives its primary input, layer V sends a non‐reciprocal, feedforward projection to higher‐order thalamic nuclei, such as the dorsal division of the MGB (Bajo et al., 1995). The layer V projection is characterized by larger axons and terminals than those descending from layer VI, and is thought to provide a driver (or Class 1) input, which can define the response characteristics of the subcortical target neurons, as opposed to the modulatory (or Class 2) influence of the layer VI projection (Lee & Sherman, 2012; Llano & Sherman, 2009; Mease, Sumser, Sakmann & Groh, 2016; Sherman & Guillery, 2001). Corticothalamocortical circuits originating from layer V therefore provide a potential substrate for information transfer between different cortical areas (Llano & Sherman, 2008; Theyel, Llano & Sherman, 2010).

Layer V neurons in the somatosensory cortex that project to the dorsal part of Po in the thalamus also send collaterals to brainstem areas, such as the anterior pretectal nucleus, the deep layers of the SC and the pontine nuclei (Veinante, Lavallée & Deschênes, 2000), providing these regions with information about ongoing cortical states. Based on the finding that layer V neurons in sensory cortical areas have these branching patterns and display complex receptive fields (Atencio & Schreiner, 2010; Martinez et al., 2005; Niell & Stryker, 2008), Guillery and Sherman (2011) proposed that they convey sensorimotor signals to multiple brain regions, including (via the thalamus) higher‐level cortical areas. Although little is known about the function of these descending projections, clues are beginning to emerge from studies in which the activity of layer V corticofugal neurons is selectively manipulated.

In the auditory cortex, layer V projects to the contralateral cortex, non‐lemniscal regions of the MGB (Bajo et al., 1995; Winer, Diehl & Larue, 2002), the striatum and various subthalamic targets, including the IC (Bajo & Moore, 2005; Bajo et al., 2007; Moriizumi & Hattori, 1991), SOC and dorsal CN (Jacomme et al., 2003). Studies in which retrograde tracers were injected into different target nuclei have shown that only a small percentage of auditory corticofugal neurons are double‐labelled (Doucet, Molavi & Ryugo, 2003; Games & Winer, 1988; Lee, Kishan & Winer, 2011). However, there are technical limitations with this approach, and there is a growing consensus that layer V neurons may broadcast signals to multiple targets (reviewed by Schofield, 2011).

**Figure 7:**
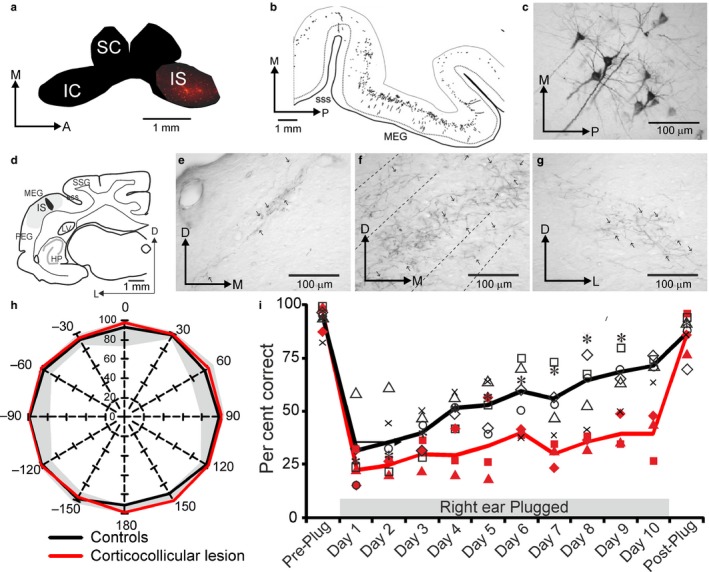
The corticocollicular pathway in the ferret. Example of a retrograde fluorescent tracer injection (IS) in the IC (a) to label cells in layer V of the auditory cortex. (b) Panoramic view and (c) photomicrograph of retrogradely labelled cells in layer V. (d) Injection of an anterograde tracer in the MEG, where the primary auditory cortex is located, produced labelled terminal fields in different regions of the ipsilateral inferior colliculus: lateral cortex (e), and dorsal cortex and central nucleus (f), as well as to a much lesser extent in corresponding regions of the contralateral IC (g). Removal of layer V cortical cells projecting to the IC by chromophore‐targeted laser photolysis does not affect the accuracy of sound localization by adult ferrets (h), but does reduce their capacity to adapt with daily training to the altered spatial cues produced by plugging one ear (i). Modified with permission from Bajo et al. (2007), Bajo, Nodal, Moore, et al. (2010).

Most attention has focussed on the corticocollicular projection, which, in ferrets, arises mostly, but not exclusively, from the primary auditory cortical areas and predominantly targets the dorsomedial region of the ipsilateral IC, including the dorsal cortex, the dorsomedial part of the central nucleus and the lateral cortex (Bajo et al., 2007) (Figure 7a–g). This innervation is broadly the same in other species, such as mice, where most of the corticollicular terminals lie within the IC's shell regions (Barnstedt, Keating, Weissenberger, King & Dahmen, 2015). Interest in the role of this descending pathway largely stems from the observation that electrical stimulation or inactivation of cortical neurons can modify almost every aspect of the response properties of IC neurons, including their sensitivity to sound frequency, intensity and location (Luo, Wang, Kashani & Yan, 2008; Ma & Suga, 2005; Nakamoto, Jones & Palmer, 2008; Zhou & Jen, 2005).

Using chromophore‐targeted laser photolysis, we set out to examine the behavioural consequences of eliminating a substantial proportion of the ipsilateral auditory corticocollicular projection in adult ferrets that had been trained to perform the 12‐loudspeaker sound localization task described earlier (Bajo, Nodal, Moore & King, 2010). Inducing apoptosis in this population of corticocollicular neurons had no effect on the accuracy with which the animals localized sounds in the horizontal plane, but did impair their ability to relearn to localize sound accurately after altering the spatial cues available by reversibly occluding one ear (Figure 7h,i). These findings therefore suggested that the corticocollicular projection has a specific role in retraining the auditory system. The publication of this study led to several discussions with Ray Guillery about the nature of the signals conveyed by these axons to the IC, which can now be addressed through *in vivo* 2‐photon imaging (Barnstedt et al., 2015), and whether the behavioural effects we observed might instead be mediated by the branches of the layer V neurons that target other structures. This is supported by the observation that sound frequency‐specific potentiation of corticostriatal synapses occurs during training on an auditory discrimination task (Xiong, Znamenskiy & Zador, 2015). But Ray was intrigued by the possibility that corticocollicular neurons send collaterals to the thalamus and in 2011 wrote to ask whether we might consider looking at the role of transthalamic corticocortical connections in auditory processing, which we are now doing.

### The thalamic reticular nucleus and intrathalamic processing

While in Oxford, Ray Guillery encouraged his colleague John Crabtree, to study the organization of the TRN. This led to the demonstration that TRN is topographically organized, with modality‐specific sectors, and that it is part of a disynaptic pathway that connects different dorsal thalamic nuclei (Crabtree, Collingridge & Isaac, 1998; Crabtree & Isaac, 2002). This last point is of major relevance because, with a few exceptions, such as the intralaminar nuclei (Smith, Bartlett & Kowalkowski, 2006), dorsal thalamic nuclei do not connect with each other directly (Jones, 1995). These studies therefore provided the first evidence for intrathalamic pathways that allow interactions between nuclei involved in different forms of sensory and motor processing. More recently, evidence has emerged that the TRN provides a substrate for interconnections in the thalamus between different sensory modalities (Paul & Llano, 2017), suggesting that these TRN‐based pathways might represent a canonical scheme for interactions between dorsal thalamic nuclei.

A potential role for the TRN in modulating information processing in the sensory thalamus has been strengthened by the finding in monkeys that it is diffusely innervated by prefrontal cortical areas (Zikopoulos & Barbas, 2006). Moreover, the ability of mice to perform a multisensory divided attention task has been shown to be dependent on interactions between the prefrontal cortex and the TRN, with this circuit mediating reductions in LGN gain, and therefore in the signals transmitted to visual cortex, when attention is directed towards auditory rather than visual stimuli (Wimmer et al., 2015). In addition to providing support for Francis Crick's “searchlight hypothesis”—that, through selective inhibition, the TRN helps to establish the focus of attention (Crick, 1984)—these studies have identified a subcortical circuit through which crosstalk between cortical areas belonging to different sensory modalities can take place, which our own laboratory is currently investigating (Lohse, Dahmen, Bajo, Mann & King, 2017).

## Context‐dependent modulation of thalamic sensory processing

Finally, we would like to share some thoughts on the role of the thalamus in sensory processing, including during active behaviour. The classical textbook description of this region as a simple relay station has for the most part been abandoned, due in large part to the findings and insights of Ray Guillery, as well as the subsequent research that his work has inspired. However, the question of what exactly the sensory thalamus does is still debated (without even getting started on the function of non‐sensory thalamus).

We believe that current evidence supports a role for the sensory thalamus as a dynamically modulated filter, using its wide‐ranging inputs to contextualize and highlight incoming information—whether it be to prioritize one sensory modality over another or a specific sensory feature—before it reaches the cortex. This allows the sensory thalamus to rapidly and efficiently set the stage for the cortical processing that subsequently takes place, and hence to help determine what kind of behaviour will follow.

**Figure 8:**
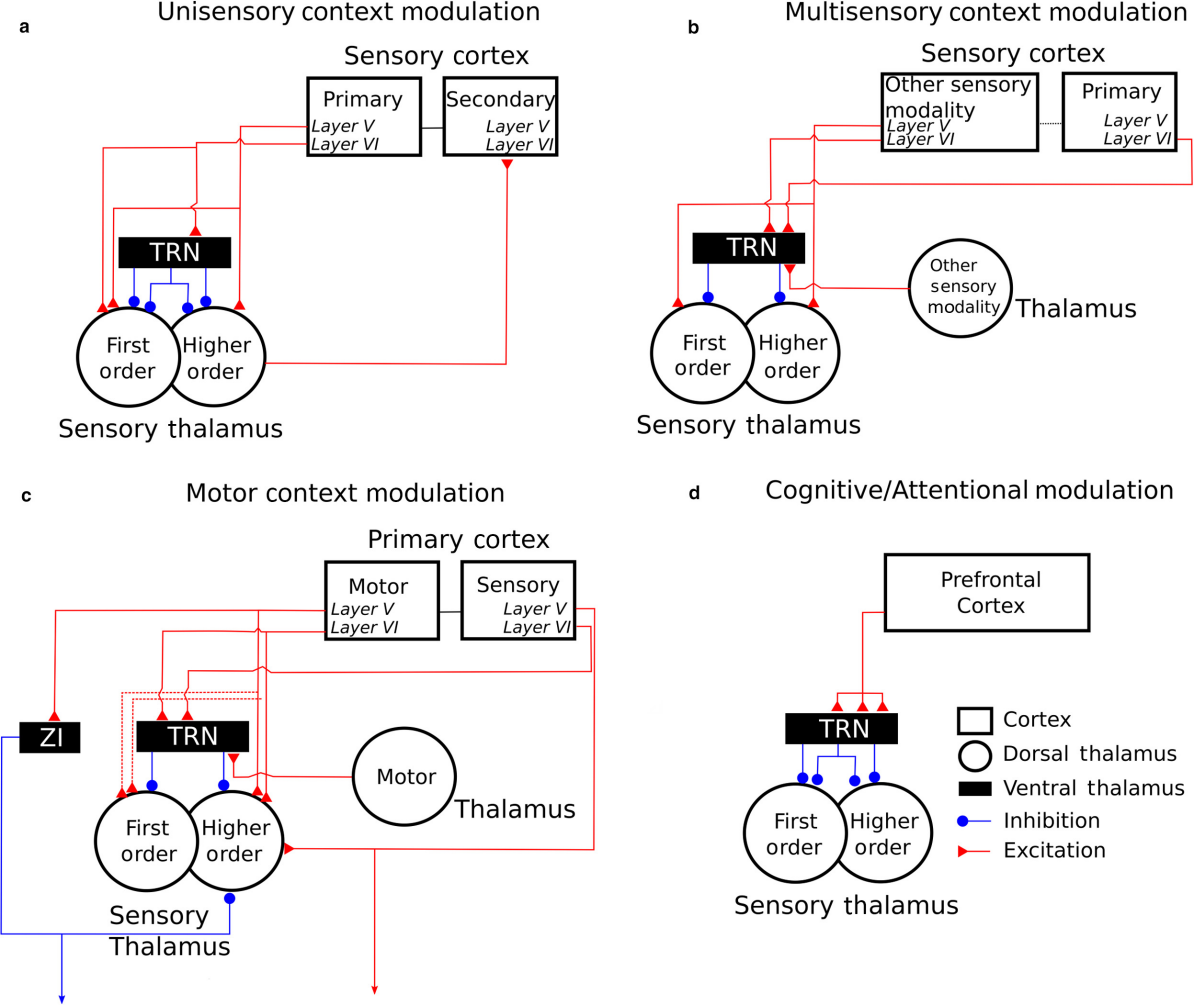
Schematic of known corticothalamic and intrathalamic projections to first‐ and higher‐order sensory thalamus, showing the nature of the interactions at each level.

As in the midbrain, electrical stimulation studies have shown that corticofugal modulation can alter the receptive field properties of neurons in the auditory thalamus (Tang, Yang & Suga, 2012; Zhang & Suga, 2000). Furthermore, recent work has shown that the receptive fields of neurons in the visual sector of cat TRN (i.e., the perigeniculate nucleus) are comparable to those of LGN neurons (Soto‐Sánchez, Wang, Vaingankar, Sommer & Hirsch, 2017). If this proves to be a general feature of TRN neurons, it suggests that descending projections operating either directly on the dorsal thalamus or via the TRN may influence the transmission of information about specific sensory features (Figure 8a).

Preliminary evidence for multisensory interactions involving layer V corticothalamic projections and connectivity between different thalamic nuclei via the TRN (Lohse et al., 2017; Paul & Llano, 2017) highlights another opportunity for context‐dependent modulation of sensory information processing (Figure 8b). Given that inputs from different sensory modalities converge at many subcortical and cortical sites, demonstrating the behavioural significance of cross‐modal influences on neural activity in the sensory thalamus will be challenging. However, Komura, Tamura, Uwano, Nishijo and Ono (2005) showed that visual cues modulate the responses of neurons in higher‐order auditory thalamus in rats in ways that correlate with the animals’ performance on an auditory discrimination task, indicating that this may be an important site for integration of multisensory cues.

An additional example of contextual modulation of activity in the sensory thalamus was highlighted by Sherman and Guillery (2001), when they proposed the possible sensorimotor nature of layer V corticothalamic projections. However, it is not only sensory cortex that returns information to sensory thalamus. Neurons in both layers V and VI of primary motor cortex provide direct input to higher‐order somatosensory thalamus, as well as suggested projections to the first‐order VPN (Urbain & Deschênes, 2007; Yamawaki & Shepherd, 2015). Another circuit for motor influences is provided by the disynaptic connections via the TRN between motor and sensory nuclei of the thalamus (Crabtree & Isaac, 2002), which, in turn, are modulated by layer VI of sensory cortex. These intrathalamic and corticothalamic inputs from motor centres provide multiple excitatory and inhibitory pathways by which sensory thalamus (in this case, the somatosensory thalamus) could be influenced during behaviour (Figure 8c).

Finally, neural circuit dynamics change during behaviour and there is growing evidence for the importance of top‐down inputs from frontal cortical areas, which has so far focused primarily on the influence of frontal cortex on processing in early sensory cortical areas (e.g., Rodgers & DeWeese, 2014; Winkowski et al., 2018). Recently, it has been shown that thalamic circuits are also recruited in a task‐dependent fashion by frontal areas of cortex associated with higher cognitive functions, providing, for example, a possible substrate for selecting between conflicting sensory stimuli (Ahrens et al., 2015; Halassa & Kastner, 2017; Wimmer et al., 2015) (Figure 8d). How these top‐down cognitive influences interact with sensory and motor corticothalamic and intrathalamic circuits remains to be determined, but the opportunity afforded by the circuit manipulation tools developed over the last 15 years will allow the ideas formulated by Ray Guillery to be put to the test.

## Conflict of Interest

The authors have no conflict of interest to declare.

## Author contributions

ML, VMB and AJK contributed equally to writing this review article.

**Abbreviations**
A1: primary auditory cortex
AAF: anterior auditory field
ADF: anterior dorsal field
AEG: anterior ectosylvian sulcus
ALLS: anterolateral lateral suprasylvian area
AMLS: anteromedial lateral suprasylvian area
aPSSC: anterior pseudosylvian sulcal cortex
AVF: anterior ventral field
C: caudal
CN: cochlear nucleus
D: dorsal
HCT: harmonic complex tone
HP: hippocampus
IC: inferior colliculus
ILD: interaural level difference
IS: injection site
ITD: interaural time difference
LGN: lateral geniculate nucleus
L: lateral
LRSS: lateral bank of the rostral suprasylvian sulcus
LV: lateral ventricle
MEG: middle ectosylvian sulcus
MGB: medial geniculate body
MGBv: medial geniculate body, ventral division
M: medial
MRSS: medial bank of the rostral suprasylvian sulcus
PEG: posterior ectosylvian sulcus
PLLS: posterolateral lateral suprasylvian area
Po: posterior group of the thalamus
PPc: caudal posterior parietal field
PPF: posterior pseudosylvian field
P: posterior
PPr: rostral posterior parietal field
pPSSC: posterior pseudosylvian sulcal cortex
PSF: posterior suprasylvian field
PS: posterior suprasylvian visual field
pss: pseudosylvian sulcus
RC: rhinal cortex
SC: superior colliculus
SIII: third somatosensory cortical area
SI: primary somatosensory cortex
SMI_32_: nonphosphorylated epitope in neurofilament H protein
SOC: superior olivary complex
SSG: suprasylvian gyrus
sss: suprasylvian sulcus
SSY: suprasylvian sulcal visual field
TRN: thalamic reticular nucleus
VPN: ventral posterior nucleus
VP: ventral posterior field
V: ventral
wm: white matter
ZI: zona incerta
