# Supplementary material for: Development, organization and plasticity of auditory circuits: Lessons from a cherished colleague
Source: Eur J Neurosci. 2018 Aug 16;49(8):990–1004. doi: 10.1111/ejn.13979 (PMC6519211; doi:10.1111/ejn.13979)
Supplement: Supplementary file 1 [file EJN-49-990-s001.pdf]

EJN European Journal of Neuroscience FENS Federation of European Neuroscience Societies

Development, Organization and Plasticity of Auditory Circuits: Lessons from a Cherished Colleague

Michael Lohse, Victoria M. Bajo and Andrew J. King

Review timeline:	Submission date:	27 February 2018
	Editorial Decision:	09 April 2018
	Revision Received:	11 May 2018
	Accepted:	23 May 2018

Editor: Paul Bolam
Reviewer 1: Daniel Llano (University of Chicago, USA)
Reviewer 2: Brett Schofield (Northeast Ohio Medical University, USA)
Reviewer 3: David Moore (Cincinnati Children's Hospital Medical Center, USA)
Reviewer 4: Edward Bartlett (Purdue University, USA)

1st Editorial Decision 09 April 2018

Dear Andy,

You paper has been reviewed at by four(!) external reviewers and the editorial team. We are pleased to say that each of the reviewers consider it to be a nicely written piece and fitting contribution to this Special Issue in honour of Ray. Each of them raise a few minor issues that we think will be easy to address, mostly by simply clarifying the text. Please carefully address each of the points they raise in a revised version.

We also noted the following points that should be attended to in the revised version.

- The figures should not be embedded in the text
- Higher resolution figures will be needed for publication
- Fig 5d needs a scale bar
- Fig 7: scale bar needed for panel c and measurements needed for scale bars in e-g
- Author contributions need to be included in the manuscript itself
- Please suppy a list of abbreviations

Thank you for your support of this Special Issue of EJN in honour of Ray.

Best wishes,

Paul & John
co-Editors in Chief, EJN

Reviews:

Reviewer: 1

Comments to the Author
In “Development, Organization and Plasticity of Auditory Circuits: Lessons from a Cherished Colleague”, Lohse, Bajo and King extensively their (and related) work on auditory system plasticity and organization, particularly as it relates to their groups interactions with their Oxford colleague, Ray Guillery, who recently passed away. I found this to be an excellent and appropriate homage to the great insights brought to neuroscience by Ray Guillery, while also doing an excellent job of summarizing an impressive body of work by the Oxford auditory group. Impressively, the manuscript also reviews very recent literature and attempts to synthesize this work by proposing modes of organization of the auditory thalamocortical system.
I believe that this review will be of importance to the field and have only minor critiques:
Page 5, first line: should be “carried out in”
Page 17, when describing that 10% of inputs to thalamocortical relay cells, and 90% come from descending and brainstem, the authors should

include the TRN among the 90%.
Page 21, when discussing branching of layer 5 neurons from AC to subcortical structures, the authors may consider adding this reference: (Brain Res Bull. 1991 Jul;27(1):141-4. Pyramidal cells in rat temporoauditory cortex project to both striatum and inferior colliculus. Moriizumi T1, Hattori T.)
Page 22, second line, last word, delete “the”
Page 24, sixth line, spelling error: “processing”

Reviewer: 2

Comments to the Author
This is a review paper for a special issue. The work is nicely written and illustrated. The manuscript appropriately highlights Guillery’s influence on the work conducted in the authors’ lab, and considers a broader perspective as well.

I have only a few suggestions for editing:

1. page 13, Figure 4 legend: The third sentence: “Grey arrows indicate the direction of tonotopic gradients.” Expecting that many readers may not be auditory physiologists, it may be helpful to state that the arrow points from low to high frequency regions (or some such wording to indicate these areas). This would make it easier to follow the text, for example, when it refers to different cortical areas that “share a common low-frequency border”.
2. page 20, Figure 6 legend, third line from bottom of legend: I assume that “SME” should be “SEM”?
3. page 21, first full paragraph (starting on the third line): Add “s” to “project” in first line (…, layer V projects…)
4. same paragraph: The authors cite original studies and a review paper for the point “that only a small percentage of auditory corticofugal neurons are double labelled (Games & Winer, 1988; Doucet et al., 2003; Lee et al., 2011).” They then acknowledge a newer point of view: “there is a growing consensus that layer V neurons may broadcast signals to multiple targets”. It would be appropriate to cite original studies that produced those data or a review that argued for that very point (e.g., Schofield, B. R. 2011. Central descending auditory pathways. In D. K. Ryugo, A. N. Popper, & R. R. Fay (Eds.), Springer Handbook of Auditory Research, Vol. 15, Auditory and Vestibular Efferents (pp. 261-290). New York: Springer-Verlag).
5. page 21, line 15: insert “of” (…where most of the corticocollicular terminals…)

Reviewer: 3

Comments to the Author
This paper provides a detailed review of the influence of Ray Guillery, in the interesting and contemporary context of sensory (mainly auditory) neuroscience performed at the University of Oxford from the early 1980s. It is a comprehensive and, for this reviewer who experienced the earlier part of that period, factually accurate account of Ray's many contributions. The paper is very well written and referenced and should make a valuable contribution to the literature. I particularly liked the skillful way in which the narrative worked Ray's influence into the interesting history and very contemporary and exciting overview of the research in the Oxford auditory lab. Just one general suggestion. Although this article rightly highlights the contribution of Ray Guillery who was particularly interested in thalamocortical communication, there are many other sources (e.g. frontal cortex, basal ganglia) of direct functional modulation of sensory cortex. I think it would be worth, in the abstract and a concluding paragraph, simply drawing attention to this fact to put the thalamic influence in a more accurate and informative context.

Specific suggestions

p.10 para 2 Actually the first behavioral study using ferrets in the Oxford lab examined spatial release from masking, essentially a detection task (Hine et al, Behav. Neurosci., 1994, 108, 196-205). For the sake of historical accuracy, relating to other work by Guillery, I think this paper should be cited, possibly in the same place as the Moore et al., 1999 study, since the same behavioral technique was used.

p.17 para 2 I'm a bit confused here, since the '10% or less' of synapses coming from the ascending system are contrasted with those coming from the brainstem and cortex. But in the auditory system, we would expect the ascending systems to be mainly brainstem. I can only presume this section is referring to the LGN where the primary ascending input is from the optic nerve. Please clarify.

p.20 'Layer V neurons that project to the thalamus have been shown . . .' Is this version correct?

Ahrens et al (2015), Bajo & Moore (2005), Homma et al (2017) not cited. There are probably others . . .

Reviewer: 4

Comments to the Author
The review by Lohse et al is a nice summary of Ray Guillery’s contributions to thalamocortical and corticothalamic circuits along with their influence on the large body of work on these circuits by the Oxford Auditory group and others. The writing was generally clear and well-articulated. A few remaining suggestions are listed below.

1. P4, change “provided a catalyst for” to “catalyzed”

2. P5, first line, change to “carried out”
3. P6, end of first paragraph, add “in both hearing and vision” to the end of the last sentence
4. P7, second line “frequency and direction-dependent”
5. P8, first line change to “and that topographic”
6. P10, end of first paragraph. Consider adding in a Shamma lab review here also.
7. Can the section on ferret auditory cortex and adaptive plasticity be tied a bit more to Ray’s work?
8. P15, first line of last paragraph. Murray's papers have been referred to, but he has not been directly singled out yet to this point.
9. P16, last line before the figure. Please add citations for this.
10. P17, second to last sentence. Although corticothalamic synapses are individually weak, their collective action can be very strong given their strong synaptic facilitation and metabotropic responses (von Krosigk and Mccormick 1992, 1999, Turner and Salt 1998, Bartlett and Smith 2002).
11. P21, second paragraph. Would the ferret dorsomedial ICC be part of the low frequency portion of the tonotopic region, as in rodents.
12. P21, second paragraph, last line. Does this apply to core and shell regions?
13. Fig. 8A. I think that the layer V input from Primary should be going to higher order cortex.
14. For both 8B and 8C, shouldn’t the layer VI also go to TRN?

Authors’ Response 11 May 2018

Response to editors and reviewers
Development, Organization and Plasticity of Auditory Circuits: Lessons from a Cherished Colleague
Michael Lohse, Victoria M. Bajo, and Andrew J. King

We are grateful to the reviewers for their positive comments. Changes to the manuscript are indicated in red font.

Editorial comments
- The figures should not be embedded in the text
- Higher resolution figures will be needed for publication
- Fig 5d needs a scale bar
- Fig 7: scale bar needed for panel c and measurements needed for scale bars in e-g
- Author contributions need to be included in the manuscript itself
- Please suppy a list of abbreviations

The embedded figures have been removed from the text and are included as separate high resolution images. Scale bars have been added to Figure 5D and Figure 7C. The values to which all scale bars correspond have been added to the figure panels, and this information deleted from the legends. Author contributions have been added to the manuscript. A list of abbreviations is now included, and these details removed from the figure legends.

Reviewer 1
Comments to the Author
In “Development, Organization and Plasticity of Auditory Circuits: Lessons from a Cherished Colleague”, Lohse, Bajo and King extensively their (and related) work on auditory system plasticity and organization, particularly as it relates to their groups interactions with their Oxford colleague, Ray Guillery, who recently passed away. I found this to be an excellent and appropriate homage to the great insights brought to neuroscience by Ray Guillery, while also doing an excellent job of summarizing an impressive body of work by the Oxford auditory group. Impressively, the manuscript also reviews very recent literature and attempts to synthesize this work by proposing modes of organization of the auditory thalamocortical system.
I believe that this review will be of importance to the field and have only minor critiques:

Page 5, first line: should be “carried out in”
“Carried in” has been corrected to “carried out in” (now top of page 7).

Page 17, when describing that 10% of inputs to thalamocortical relay cells, and 90% come from descending and brainstem, the authors should include the TRN among the 90%.
This has been clarified as suggested (and to stress that it refers to the visual system), so that the sentence now reads (now page 15):

“Indeed, studies in the visual system have revealed that only around 10% or less of the synapses on these relay neurons come from their ascending sensory inputs, with the rest being predominantly of either cortical, ventral thalamus, or brainstem origin (Van Horn et al., 2000; Guillery & Sherman, 2011).”

Page 21, when discussing branching of layer 5 neurons from AC to subcortical structures, the authors may consider adding this reference: (Brain Res Bull. 1991 Jul;27(1):141-4. Pyramidal cells in rat temporoauditory cortex project to both striatum and inferior colliculus. Moriizumi T1, Hattori T.)
Reference added, as suggested (now page 18):
"In the auditory cortex, layer V projects to the contralateral cortex, non-lemniscal regions of the MGB (Bajo et al., 1995; Winer et al., 2002), the striatum and various subthalamic targets, including the IC (Moriizumi & Hattori, 1991; Bajo & Moore, 2005, Bajo et al., 2007), SOC and dorsal CN (Jacomme et al., 2003)."

Page 22, second line, last word, delete "the"
Corrected.

Page 24, sixth line, spelling error: "processing"
Corrected (now page 20).

Reviewer 2
Comments to the Author
This is a review paper for a special issue. The work is nicely written and illustrated. The manuscript appropriately highlights Guillery's influence on the work conducted in the authors' lab, and considers a broader perspective as well.

I have only a few suggestions for editing:

1. page 13, Figure 4 legend: The third sentence: "Grey arrows indicate the direction of tonotopic gradients." Expecting that many readers may not be auditory physiologists, it may be helpful to state that the arrow points from low to high frequency regions (or some such wording to indicate these areas). This would make it easier to follow the text, for example, when it refers to different cortical areas that "share a common low-frequency border".
We agree and have added a sentence in parentheses stating that tonotopic gradients are illustrated as low to high frequency.

2. page 20, Figure 6 legend, third line from bottom of legend: I assume that "SME" should be "SEM"?
Yes, corrected (thank you)

3. page 21, first full paragraph (starting on the third line): Add "s" to "project" in first line (…, layer V projects…)
Corrected (page 18).

4. same paragraph: The authors cite original studies and a review paper for the point "that only a small percentage of auditory corticofugal neurons are double labelled (Games & Winer, 1988; Doucet et al., 2003; Lee et al., 2011)." They then acknowledge a newer point of view: "there is a growing consensus that layer V neurons may broadcast signals to multiple targets". It would be appropriate to cite original studies that produced those data or a review that argued for that very point (e.g., Schofield, B. R. 2011. Central descending auditory pathways. In D. K. Ryugo, A. N. Popper, & R. R. Fay (Eds.), Springer Handbook of Auditory Research, Vol. 15, Auditory and Vestibular Efferents (pp. 261-290). New York: Springer-Verlag).
We agree and apologise for this omission. The suggested reference has been added (now page 18):
"However, there are technical limitations with this approach and there is a growing consensus that layer V neurons may broadcast signals to multiple targets (reviewed by Schofield, 2011)."

5. page 21, line 15: insert "of" (…where most of the corticocollicular terminals…)
Corrected (page 18).

Reviewer 3:
Comments to the Author
This paper provides a detailed review of the influence of Ray Guillery, in the interesting and contemporary context of sensory (mainly auditory) neuroscience performed at the University of Oxford from the early 1980s. It is a comprehensive and, for this reviewer who experienced the earlier part of that period, factually accurate account of Ray's many contributions. The paper is very well written and referenced and should make a valuable contribution to the literature. I particularly liked the skillful way in which the narrative worked Ray's influence into the interesting history and very contemporary and exciting overview of the research in the Oxford auditory lab. Just one general suggestion. Although this article rightly highlights the contribution of Ray Guillery who was particularly interested in thalamocortical communication, there are many other sources (e.g. frontal cortex, basal ganglia) of direct functional modulation of sensory cortex. I think it would be worth, in the abstract and a concluding paragraph, simply drawing attention to this fact to put the thalamic influence in a more accurate and informative context.
We agree with the point that higher-order cortical areas also affect early sensory cortical processing, especially during behaviour. The last paragraph of the concluding section (now page 22) highlights the need to consider the influence of frontal cortical areas on thalamic processing. We have expanded this paragraph to mention the role of corticocortical interactions too (and added two new references). We disagree, however, with changing the abstract since this is a small component of the article and doesn't really fit with the points that are made there.
"Finally, neural circuit dynamics change during behavior and there is growing evidence for the importance of top-down inputs from frontal

cortical areas, which has so far focused primarily on the influence of frontal cortex on processing in early sensory cortical areas (e.g. Rodgers & DeWeese, 2014; Winkowski et al., 2018). Recently, it has been shown that thalamic circuits are also recruited in a task-dependent fashion by frontal areas of cortex associated with higher cognitive function, providing, for example, a possible substrate for selecting between conflicting sensory stimuli (Ahrens et al., 2015; Wimmer et al., 2015; Halassa & Kastner, 2017) (Fig. 8D). How these top-down cognitive influences interact with sensory and motor corticothalamic and intrathalamic circuits remains to be determined, but the opportunity afforded by the circuit manipulation tools developed over the last 15 years will allow the ideas developed by Ray Guillery to be put to the test."

Specific suggestions
p.10 para 2 Actually the first behavioral study using ferrets in the Oxford lab examined spatial release from masking, essentially a detection task (Hine et al, Behav. Neurosci., 1994, 108, 196-205). For the sake of historical accuracy, relating to other work by Guillery, I think this paper should be cited, possibly in the same place as the Moore et al., 1999 study, since the same behavioral technique was used.
Agreed and this reference has been added.
"Long-term occlusion of one ear, initiated either during infancy or in adulthood, was found to reduce the ability of ferrets to detect a tone in the presence of masking noise originating from other directions, with levels of "binaural unmasking" gradually recovering after normal binaural inputs were restored (Hine et al., 1994; Moore et al., 1999)."

p.17 para 2 I'm a bit confused here, since the '10% or less' of synapses coming from the ascending system are contrasted with those coming from the brainstem and cortex. But in the auditory system, we would expect the ascending systems to be mainly brainstem. I can only presume this section is referring to the LGN where the primary ascending input is from the optic nerve. Please clarify.
Yes, it relates to the visual system. This has been clarified along with the change requested by reviewer 1 (now page 15):
"Indeed, studies in the visual system have revealed that only around 10% or less of the synapses on these relay neurons come from their ascending sensory inputs, with the rest being predominantly of either cortical, ventral thalamus, or brainstem origin (Van Horn et al., 2000; Guillery & Sherman, 2011)."

p.20 'Layer V neurons that project to the thalamus have been shown . . .' Is this version correct?
Yes, but since there seems to be some confusion, we have expanded this sentence to provide more detail (top of page 18):
"Layer V neurons in the somatosensory cortex that project to the dorsal part of Po in the thalamus also send collaterals to brainstem areas, such as the anterior pretectal nucleus, the deep layers of the SC and the pontine nuclei (Veinante et al., 2000), providing these regions with information about ongoing cortical states."

Ahrens et al (2015), Bajo & Moore (2005), Homma et al (2017) not cited. There are probably others .
Thank you, we have added the missing references.

Reviewer 4
Comments to the Author
The review by Lohse et al is a nice summary of Ray Guillery's contributions to thalamocortical and corticothalamic circuits along with their influence on the large body of work on these circuits by the Oxford Auditory group and others. The writing was generally clear and well-articulated. A few remaining suggestions are listed below.

1. P4, change "provided a catalyst for" to "catalyzed"
We have changed this, but prefer the following wording (now page 6):
"This paper led to several other groups adopting this species as a model for investigating the…"

2. P5, first line, change to "carried out"
Corrected (now page 7).

3. P6, end of first paragraph, add "in both hearing and vision" to the end of the last sentence
We have changed this, but usually slightly different (but equivalent) wording (now page 8):

"The physiological changes that occur following unilateral hearing loss therefore again support the concept of competitive interactions between the two sense organs in both the visual and auditory systems."

4. P7, second line "frequency and direction-dependent"
This change would not be correct, since the sentence refers to the amplitude spectrum, which already incorporates amplitude and frequency. No change has therefore been made.

5. P8, first line change to "and that topographic"
Change made as suggested (now page 9).

6. P10, end of first paragraph. Consider adding in a Shamma lab review here also.
We have added Fritz et al. (2007) (now page 10).

7. Can the section on ferret auditory cortex and adaptive plasticity be tied a bit more to Ray's work?

Not easily. We certainly had this in mind when writing it, but we don't think there is an easy way of mentioning Ray without making it sound too contrived. This section does lie naturally between Ray's developmental studies and his work on descending projections, so we think it fits well between these sections. Since none of the other reviewers commented on this, we would prefer to leave it as it is.

8. P15, first line of last paragraph. Murray's papers have been referred to, but he has not been directly singled out yet to this point.
We have left this unchanged, as we wanted to stress that the work on thalamocortical interactions is another example of their very productive collaboration. We previously referred to several earlier papers from the Guillery lab on the development of the visual pathways. Murray was obviously involved in several of these (particularly the behavioural and electrophysiological characterization of the effects of eyelid suture). We have therefore amended a sentence at the top of page 8 to mention Murray more directly:
"Moving beyond their predominantly anatomical approach, Ray Guillery and his colleagues, particularly Murray Sherman, adopted behavioural and electrophysiological measures to show in cats that responses to stimulation of a previously deprived eye are largely eliminated within the binocular regions of the visual pathways (Sherman et al., 1974)."

9. P16, last line before the figure. Please add citations for this.
We have added Sherman and Guillery (2001) to the end of this sentence (now page 15).

10. P17, second to last sentence. Although corticothalamic synapses are individually weak, their collective action can be very strong given their strong synaptic facilitation and metabotropic responses (von Krosigk and Mccormick 1992, 1999, Turner and Salt 1998, Bartlett and Smith 2002).
We have added a sentence (plus new references) to address this important point (now page 15):
"Measurements of the postsynaptic responses evoked by activation of corticothalamic synapses suggests that these signals are likely to have a pronounced effect on the transmission of information through the thalamus (McCormick & Von Krosigk, 1992; von Krosigk et al., 1999; Turner & Salt, 1998; Bartlett & Smith, 2002)."

11. P21, second paragraph. Would the ferret dorsomedial ICC be part of the low frequency portion of the tonotopic region, as in rodents.
Yes, probably. We have rewritten this to avoid any confusion (now page 18):
"Most attention has focussed on the corticocollicular projection, which, in ferrets, arises mostly, but not exclusively, from the primary auditory cortical areas and predominantly targets the dorsomedial region of the ipsilateral IC, including the dorsal cortex, the dorsomedial part of the central nucleus and the lateral cortex (Bajo et al., 2007) (Fig. 7A-G)."

12. P21, second paragraph, last line. Does this apply to core and shell regions?
Yes, it does, though this information is not always reported, so we think it safer to keep the wording as it is.

13. Fig. 8A. I think that the layer V input from Primary should be going to higher order cortex.
True, this has been added.

14. For both 8B and 8C, shouldn't the layer VI also go to TRN?
This has been added.
